# Martini 3 Coarse-Grained Model for the Cofactors Involved in Photosynthesis

**DOI:** 10.3390/ijms25147947

**Published:** 2024-07-20

**Authors:** Maria Gabriella Chiariello, Rubi Zarmiento-Garcia, Siewert-Jan Marrink

**Affiliations:** Groningen Biomolecular Sciences and Biotechnology Institute, University of Groningen, Nijenborgh 7, 9747 AG Groningen, The Netherlands; m.g.chiariello@rug.nl (M.G.C.); r.zarmiento.garcia@rug.nl (R.Z.-G.)

**Keywords:** light-harvesting cofactors, coarse-graining (CG), Martini 3, molecular dynamics simulations, photosystem II, CG parametrization, free energy calculations, protein dynamics

## Abstract

As a critical step in advancing the simulation of photosynthetic complexes, we present the Martini 3 coarse-grained (CG) models of key cofactors associated with light harvesting (LHCII) proteins and the photosystem II (PSII) core complex. Our work focuses on the parametrization of beta-carotene, plastoquinone/quinol, violaxanthin, lutein, neoxanthin, chlorophyll A, chlorophyll B, and heme. We derived the CG parameters to match the all-atom reference simulations, while structural and thermodynamic properties of the cofactors were compared to experimental values when available. To further assess the reliability of the parameterization, we tested the behavior of these cofactors within their physiological environments, specifically in a lipid bilayer and bound to photosynthetic complexes. The results demonstrate that our CG models maintain the essential features required for realistic simulations. This work lays the groundwork for detailed simulations of the PSII-LHCII super-complex, providing a robust parameter set for future studies.

## 1. Introduction

Photosystem II (PSII) is a crucial component of the photosynthetic apparatus in plants, algae, and cyanobacteria, playing a fundamental role in the initial stages of the photosynthetic process [1,2,3]. This large protein super-complex is responsible for the capture and conversion of light energy into chemical energy, a process essential for the sustenance of life on Earth. PSII operates in the thylakoid membrane of chloroplasts, where it utilizes light-harvesting antenna proteins (LHCs) to efficiently collect photons from sunlight and funnel the excitation energy toward the reaction center [4,5,6]. The reaction center of PSII performs the critical task of splitting water molecules into molecular oxygen, protons, and electrons. This water-splitting reaction not only generates the oxygen we breathe but also provides the electrons necessary for the subsequent steps of the photosynthetic electron transport chain [7,8]. The functionality of PSII is heavily dependent on various cofactors, including chlorophylls, carotenoids, and quinones, which play pivotal roles in light absorption, energy transfer, and electron transport [9,10]. Additionally, the major light-harvesting complex (LHCII), mainly associated with PSII, consists of pigment–protein complexes that optimize photon capture and contribute to the photoprotection mechanisms under high light conditions [11,12,13]. These sophisticated arrangements of proteins and cofactors ensure the efficiency and stability of the photosynthetic process, highlighting the intricate and highly evolved nature of PSII-LHCII super-complex as a vital player in the conversion of solar energy into chemical energy. 

Recent high-resolution structures of the entire PSII-LHCII super-complex obtained through techniques such as cryo-electron microscopy (cryo-EM) have revealed detailed information about the arrangement and interactions of the various protein components within the photosynthetic machinery [14,15]. The availability of high-resolution structures paves the way for molecular dynamics (MD) simulation studies, enabling to potentially investigate the dynamics of the LHCII-PSII super-complex with remarkable detail. These simulations can be performed both at all-atom (AA) resolution and using coarse-grained (CG) models, offering insights into the structural flexibility, stability, and functional mechanisms of both the individual and super-complexes [16,17,18,19,20,21,22]. Whereas AA models offer the highest accuracy, the benefit of CG models is the larger spatiotemporal scales that can be assessed.

In the past, several efforts have employed CG models based on the Martini 2 force field to study the PSII and LHCII complexes, along with their associated cofactors in the thylakoid membrane [23,24,25,26]. These CG models have proven invaluable for investigating various aspects of photosynthetic machinery due to their efficiency in simulating large biomolecular systems over extended timescales. For example, this approach has been particularly useful in exploring the exchange pathways of plastoquinone and plastoquinol within PSII [25]. The simulations have revealed detailed mechanisms of how these molecules move through the complex protein environment, which is critical for the efficient transfer of electrons during the photosynthetic process. Additionally, a Martini-based CG approach was used to unveil the lipid composition surrounding LHCII in its monomeric and trimeric forms, as well as around the PSII complex, providing insights into the lipid fingerprint of the thylakoid membrane [27]. 

In parallel, the recent development of the Martini 3 force field has led to significant improvements in the CG representation of proteins, lipids, and small molecules, thanks to the introduction of new bead types and rescaled interactions, further refining the accuracy and reliability of Martini-based CG simulations [28]. For example, this enhanced force field has demonstrated its efficacy in capturing the complex interplay between small molecules and proteins, enabling study at larger timescales of processes like molecular recognition, binding events, and structural rearrangement [29]. In addition, Martini 3 has been also applied to study the pH-dependent behavior of the PsbS protein, involved in photosynthesis regulation [30].

As an initial step towards the ambitious challenge of simulating the entire PSII-LHCII super-complex in the thylakoid membrane with the updated Martini 3 force field, here, we present the Martini 3 CG parameterization of the cofactors associated with LHCII proteins and the core complex of PSII. In particular, we considered the following cofactors: beta-carotene, violaxanthin, neoxanthin, lutein, plastoquinone/quinol, chlorophyll A, chlorophyll B, and the heme group. The models were validated against pre-existing all-atom GROMOS reference simulations [31], regarding bonded and volumetric parameters. The reference GROMOS topologies of the cofactors were derived from optimized structures, with partial charges calculated at the quantum mechanical level. These topologies were validated by comparing the experimentally available water/octanol partition coefficients. The GROMOS topologies were then employed for simulating the cofactors in both membrane and protein environments [31]. 

As a cornerstone of the Martini model, partitioning free energies from water to octanol were calculated and compared with available experimental data. Furthermore, the behavior of the cofactors is examined within their physiological environment: a lipid bilayer and the LHCII protein monomer, and the PSII-LHCII super-complex. 

This paper is structured as follows: Section 3 outlines the computational procedures and the protocol for parameterizing the CG model. In Section 2, we present the finalized topologies and detail the crucial steps in the parameterization process. Additionally, molecular dynamics simulations and analyses of beta-carotene, violaxanthin, neoxanthin, lutein, and plastoquinone in a model membrane are discussed. The dynamics of LHCII at both atomistic and coarse-grained levels are addressed in the next subsection of the Results, and finally we showcase how the new models can be integrated into the PSII-LHCII super-complex inside a realistic thylakoid membrane. Concluding remarks on future applications and limitations of the cofactor models are provided in Section 4. 

## 2. Results and Discussion

### 2.1. Parametrization 

**Figure 1:**
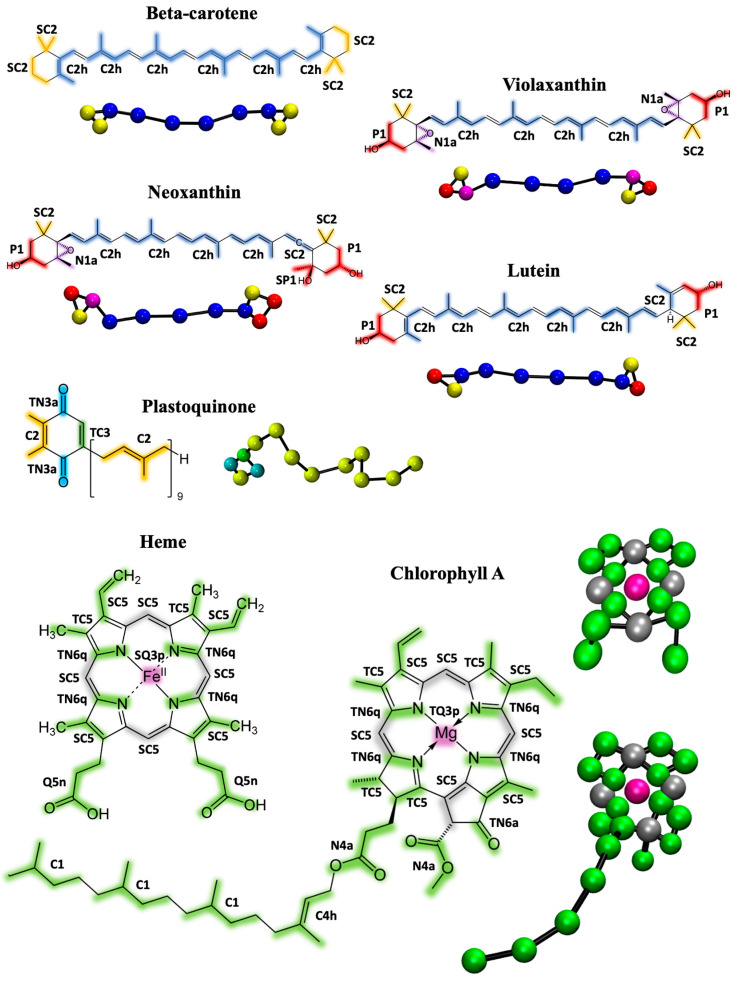
Molecular structures and CG mapping scheme of the cofactors parametrized in this work: beta-carotene, violaxanthin, neoxanthin, lutein, plastoquinone, chlorophyll A, and heme group. The atoms that are grouped into CG beads are color-coded, and the resulting CG structures in ball-and-stick representation are shown for each molecule. The size of the spheres does not reflect the beads size. Chlorophyll B has the same structure as chlorophyll A with the exception of one substituent, while plastoquinol is the reduced form of plastoquinone. The resulting CG mappings are the same and are shown in Figure S1.

The first step in the Martini 3 parametrization procedure involves virtually fragmenting the molecules and assigning them to appropriate bead types. Figure 1 illustrates the chosen mapping and bead assignments for all the cofactors involved in this study. 

Comprehensive computational details of the parametrization process are provided in Section 3. Before describing the bead assignment of the individual molecules in detail, it is important to highlight some general considerations applicable to all compounds examined in this work [32]. The mapping process determines which atoms at the AA level are represented by a single bead in the CG model. Typically, within the context of Martini 3, one can map two to six heavy atoms into a single bead. The number of atoms mapped to each bead, together with the degree of branching, dictates the bead sizes. It is generally advisable to keep functional groups intact as much as possible to preserve their chemical identity and interactions. However, for compounds that contain extensive conjugated aromatic rings, such as chlorophyll and heme, the molecules are fragmented to a greater degree. This fragmentation is necessary for an accurate representation and is facilitated by using the newly introduced tiny (T) beads, which can effectively model the smaller, more intricate segments of these molecules. The next phase involves deriving the bonded parameters, including bonds, angles, and dihedrals, for the CG models. This is accomplished by iteratively matching the distributions of bond lengths, angles, and dihedrals observed in the AA simulations, mapped into the chosen CG fragmentation. The reference AA model used in this process is based on the GROMOS force field [33], which has already been utilized for the parametrization of cofactors in the previous Martini 2 framework [31,34,35]. 

**Figure 2:**
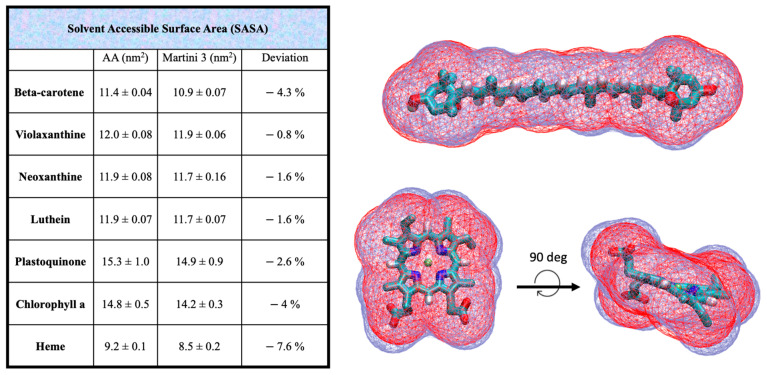
Solvent-accessible surface area (SASA) data computed at CG and AA resolution for all the cofactors parametrized in this work. The overlap of the CG (red) and AA (blue) Connolly surfaces for beta-carotene and heme are shown in the right panel. The SASA is systematically underestimated in the CG structures compared to the AA models, ranging from the −0.8% in violaxanthin to the −7.6% in the heme group.

To ensure the accuracy of the CG models, further validation was performed by comparing the solvent-accessible surface areas (SASAs) and Connolly surfaces of the AA models with their CG counterparts for all cofactors. These comparisons help verify that the CG models retain the essential structural and volumetric properties of the original AA models. The results of these validations are presented in Figure 2. 

Nonbonded interactions in the CG models are defined based on the interactions of the bead types, which depend on the specific bead assignments. In this study, the selection of bead types was guided by matching the free energies of transfer from octanol to water for each cofactor. This approach ensures that the CG models accurately reproduce the partitioning behavior of the molecules. Experimental values for the water/octanol partition coefficient (log *P*) are available for most of the cofactors; however, for plastoquinone, the predicted value obtained from the software XLogP3 (version 3.2.2) [36] is reported. The computed and experimental partition coefficients are reported in Table 1. 

*Beta-carotene*, *Violaxanthin*, *Neoxanthin*, *Lutein*, *and Plastoquinone/quinol*: Xanthophylls and carotenoids share common molecular structure; therefore, the CG fragmentation is similar and consistent. The conjugated double-bond chain connecting the two rings is divided into four parts, modelled with C2h beads using a 5:1 or 6:1 mapping. The two rings and their substituent are fragmented into three beads of varying polarity. In particular, small SC2 beads are used for the aliphatic groups (3:1 mapping), P1 beads for the alcohol groups (4:1 mapping), and N1a beads for the epoxy groups (4:1 mapping). Beta-carotene, violaxanthin, and lutein are modeled by a total of 10 beads, 11 bonds, and 8 angles as bonded parameters. Neoxanthin is more fragmented, using 11 beads, 12 bonds, and 9 angles. The CG optimized distributions and their comparison with those from the mapped AA simulations are shown in Figures S2–S8. Plastoquinone features a long double-bond chain, described by nine C2 beads, while the ring head is fragmented into three tiny beads (2:1 mapping, TN3a for the quinone groups, and TC3 for the aromatic ring bond) and one regular C2 bead. The plastoquinol, the reduced form of plastoquinone, has the same structure except for the two quinone groups that in the reduced form are modeled by TN6 beads (Figure S1). Plastoquinone/plastoquinol is therefore modeled with a total of 13 beads, 13 bonds, and 12 angles as bonded parameters. The distributions of the bonds between beads of the ring heads from the mapped AA simulations are very sharp; to optimally match the CG distributions, constraints are used in place of bonds in the topology. Four angles of the beta-carotene (associated with the linker chain) show a bimodal distribution, which the CG model cannot fully capture, resulting in an average distribution covering the two peaks. A hypothetical solution to model bimodal distributions involves deriving tabulated potentials, which unfortunately are not fully supported in recent versions of Gromacs. 

We computed and compared the solvent accessible surface areas of the AA and CG cofactors (Figure 2). The CG volume is slightly lower of the AA counterpart for all the cofactors, with an underestimation ranging from −0.8% for violaxanthin to −4.3% of the beta-carotene, which is considered acceptable. This slight volumetric underestimation is a common feature of the Martini model. The overlap of the beta-carotene Connolly surface shows indeed a good match between AA and CG models. 

The water/octanol partition coefficient of the beta-carotene (log *P*_CG_ = 17.6) computed with this bead assignment perfectly matches the literature value (log *P*_Exp_ = 17.6) [37], as does that of the violaxanthin (log *P*_CG_ = log *P*_Exp_ = 12.0) [37]. The values are listed in Table 1. Neoxanthin (log *P*_CG_ = 10.9) and lutein (log *P*_CG_ = 12.9) are slightly more hydrophilic than the experimental values (log *P*_Exp_ = 11.9 and 14.8 for neoxanthin and lutein, respectively) [37]. However, the hydrophobicity trend among the compounds is reproduced (beta-carotene > lutein > violaxanthin > neoxanthin). Plastoquinone has a computed log *P*_CG_ = 19.8; unfortunately, comparative experimental values are not available, but a prediction from the XLogP3 [36] software (version 3.2.2) gives log *P* = 20.4. Plastoquinol is slightly more hydrophilic (log *P*_CG_ = 18.6), reflecting the trend of the log *p* values for plastoquinone/quinol heads (log *P*_CG_ = 2.6 and 1.4, respectively). There is also a good correlation with the reported experimental values for tetramethyl 1,4-benzoquinones/quinol, with log *P*_Exp_ values of 2.5 and 1.8, respectively [38] (see Table 1).

**Table 1:** Log *P* values for the partitioning of Martini 3 cofactors between water and octanol.

Molecule	Martini 3	Literature
Beta-carotene	17.6 ± 0.05	17.6 ^1^
Violaxanthin	12.0 ± 0.04	12.0 ^1^
Neoxanthin	10.9 ± 0.04	11.9 ^1^
Lutein	12.9 ± 0.04	14.8 ^1^
Plastoquinone ^3^	19.8 ± 0.11	
Plastoquinol	18.6 ± 0.10	
Plastoquinone (head)	2.6 ± 0.03	2.5 ^4^
Plastoquinol (head)	1.4 ± 0.04	1.8 ^4^
Chlorophyll A	11.9 ± 0.10	2.21 ^2^
Chlorophyll B	11.2 ± 0.10	
Heme	−0.31 ± 0.04	0.95 ^2^
Chlorophyll A (aromatic core)	1.9 ± 0.07	
Chlorophyll B (aromatic core)	1.4 ± 0.05	

^1^ Value from reference [37]. ^2^ Value from reference [39]. ^3^ There are no experimental values for plastoquinone, but a there is prediction of log *P* = 20.4 from XLogP3. ^4^ The values are evaluated for tetramethyl 1,4 benzoquinones/quinol in Reference [38].

*Chlorophylls and heme:* The mapping and the bead assignment for chlorophyll A and the heme group is shown in Figure 1. Chlorophyll B has the same structure as chlorophyll A except for one ring substituent, as shown in Figure S1. The ring structure of chlorophyll is a closely packed conjugated aromatic system, which is why we extensively used tiny beads (mapping 2:1 or 1:1). The magnesium ion is represented by one TQ3p bead with a charge of +1, while the surrounding four beads of the imidazole rings are TN6q type, each carrying a partial charge of −0.25. These charges reflect those of the corresponding AA model, ensuring the total charge of the molecule is zero. The charges on the Mg and surrounding beads mimic the strong quadripolar nature of this portion of the molecule. The rest of the aromatic system is described by TC5 (2:1 mapping) and SC5 (3:1) beads. To optimally maintain the molecule’s symmetry, four atoms have been doubly mapped. Two methyl carboxylate groups are modeled with N4a beads, while the aliphatic chain is fragmented into four regular C1 beads. The CG model consists of 23 beads, resulting in 44 bonds, 22 angles, and 15 improper dihedrals. Due to the sharp AA distribution of the bonds between tiny beads, we used constraints instead of bond parameters to match the AA distributions optimally. Dihedral angles are employed to keep the ring system planar and to capture a reasonable orientation of the substituents with respect to the aromatic system. The comparison between the CG and AA bonded distributions is shown in Figures S7 and S8. 

The structure of heme is very similar to that of chlorophyll, so a similar fragmentation was applied. The heme ion, iron (Fe), is modeled by a SQ3p bead to reflect its larger size compared to magnesium. This carries a charge of 0.4, while the surrounding TN6q beads have a charge of −0.1, mirroring the charges of the AA structure. However, the molecule has a total charge of −2 because the two carboxylate moieties (described by Q5n beads, each with a charge of −1) are deprotonated at a physiological pH of 7.0. The topology of heme contains 19 beads, with the same bonds, constraints, angles, and dihedrals used for chlorophylls. The SASA (Figure 2) again shows the slight underestimation of the CG model compared to AA, with a 4% and 7.6% difference for chlorophyll and heme, respectively. The only available experimental values for the water/octanol partition coefficient log *P* (reported in Table 1) are 2.21 and 0.95 for chlorophyll A and heme, respectively [39]. The computed values of our model are 11.9 and −0.31. This is a substantial improvement compared to the Martini 2 models, where the predicted log *P* was 17.8 and 8.9 for chlorophyll A and heme, respectively [31]. However, heme is slightly more hydrophilic than the experimental value, while chlorophyll is substantially more hydrophobic compared to the experimental value. Notably, several log *P* estimations from online predictors for pheophytin, which has the same structure as chlorophyll but without the ion, predicted log *P* values between 7.2 and 12, averaging around 10.4 [31], which are close to our chlorophyll log *P* computation. Additionally, the computed log *P* of the chlorophyll ring, excluding the four beads of the hydrophobic chain, shows a value (log *P* = 1.9) close to the log *P* of the heme group. We cannot exclude the possibility that the experimental value is incorrect, especially considering the complex amphipathic nature of chlorophyll, which may complicate the experimental measurement. Finally, the log *P* of chlorophyll B is slightly more hydrophilic than chlorophyll A, reflecting the polar nature of the bead in position 15, which is SN4a instead of the TC5 in chlorophyll A (see Figure S1 and Table 1).

### 2.2. Validation in Membrane Environment 

**Figure 3:**
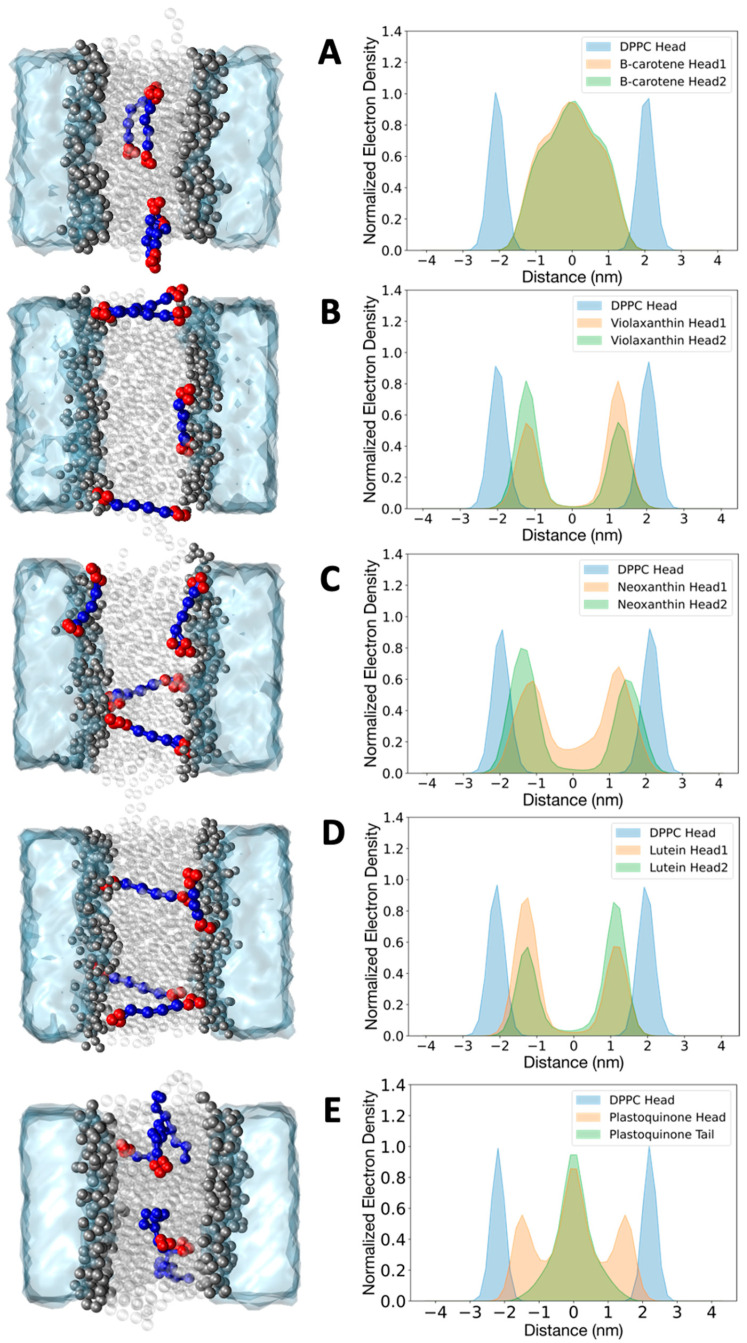
Normalized electron density plot for beta-carotene (**A**), violaxanthin (**B**), neoxanthin (**C**), lutein (**D**), and plastoquinone (**E**) in a DPPC bilayer. Densities were obtained with the coarse grained model. Densities for the DPPC head beads (blue) are reported for all the simulations. The profiles for all the cofactors are averaged over four molecules. The densities for beta-carotene, violaxanthin, lutein, and neoxanthin are shown separately for the two headgroups (green and orange). The density of plastoquinone is reported for the head group (orange) and the last bead tail (green). Representative configurations extracted from the trajectories are depicted in the left panel for each molecule.

To further validate the behavior of the CG cofactor topologies, we should consider them in their native environment. Understanding the organization and dynamics of the CG model of plastoquinone in the thylakoid membrane is crucial for studying electron transport between different protein complexes in the photosynthetic pathway [40]. Carotenoids are biosynthesized and primarily located in the membranes of plants and microorganisms, where they influence the structural and dynamic properties of the lipid membrane [41,42]. At certain concentrations, they are organized differently within the lipid membrane depending on their polarities, as observed in several studies [43,44,45]. Here, we verify that the new CG models of beta-carotene, violaxanthin, neoxanthin, lutein, and plastoquinone can capture the dynamics and organization of these cofactors in a model membrane (DPPC) based on their hydrophobicity. The choice to use DPPC as a membrane model is due to the availability of experimental studies and atomistic simulations, which we can directly compare [31,42,46,47]. The setup for the simulations was consistent across the five systems. Four molecules of each compound were placed in the DPPC bilayer. After minimization and equilibration steps, the simulations were run for 8 microseconds. The computational details of the simulations are described in Section 3. The normalized electron density plots for the head groups of carotenoids and lipids, along with a snapshot of a representative configuration from the trajectories, are shown in Figure 3.

The heads of beta-carotene showed no significant difference in the electron density profiles, remaining within the inner part of the bilayer without a preferential orientation. The molecules mostly stayed in an elongated conformation at the bilayer midplane and did not form specific clusters. This is consistent with previous studies of linear dichroism and MD simulations, which indicated that this carotenoid has an average orientation with its long axis roughly perpendicular to the lipid acyl chains [31,44,47]. The presence of more polar head groups in violaxanthin, neoxanthin, and lutein changes the electron density profiles [46]. The head groups of violaxanthin and lutein rarely lie in the hydrophobic inner part of the membrane; instead, they remain close to the polar head groups of the lipid bilayer. No clustering of the four molecules was observed. Notably, two conformations were observed in line with experimental evidence: (1) the molecule spans the membrane, exposing the two head groups to opposite bilayer leaflets, and (2) the molecule is horizontally oriented, exposing both heads to the same leaflet. The latter conformation causes the asymmetry of the two peaks in the electron density profiles of violaxanthin and lutein. Neoxanthin also reveals the presence of two relevant populations of conformations, but the two heads are not identical. In particular, the more hydrophobic head (without the epoxydic group, see Figure 1 and Figure S1), has a more significant density at the center of the membrane. In the case of plastoquinone, we present the normalized electron density profile plot for the head and the last bead of the tail. The tail bead is primarily located at the center of the bilayer, while the head is symmetrically associated with the polar heads of the bilayer. This indicates that flip-flop events, where the head group flips from one monolayer to the other, occurred, as observed in previous atomistic simulations as well as with the Martini 2 model [31]. Various conformations are also observed here, with the aliphatic chain of plastoquinone adopting what are known as U or L conformations.

### 2.3. Validation in Protein Environment 

To investigate the behavior of the cofactors in a protein environment, we selected a key component of the light-harvesting super-complex: the major light-harvesting complex II (LHCII) in its monomeric form. 

**Figure 4:**
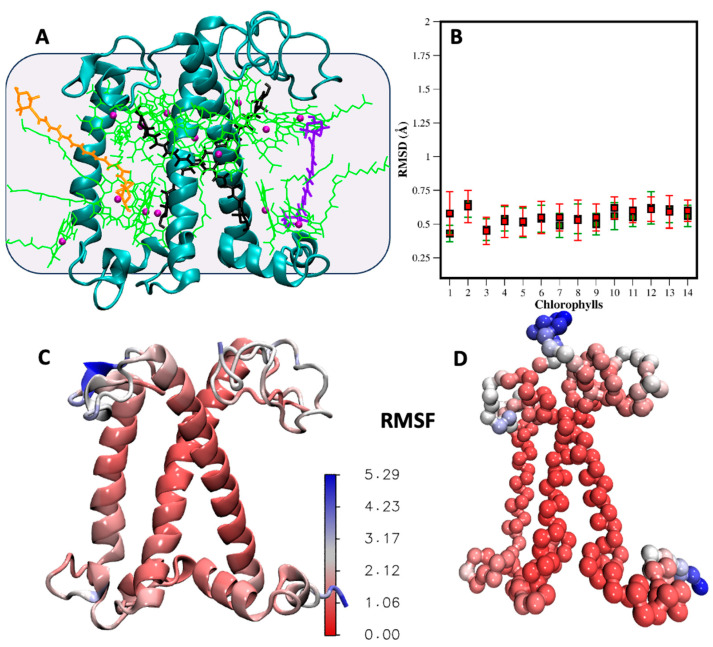
Comparison of AA and CG simulation of the LHCII monomer. (**A**) Structure of the major light-harvesting complex monomeric protein examined in this work. The protein is embedded in a POPC bilayer, which is represented as a grey background for clarity. LHCII features three transmembrane helices and binds 18 pigment molecules: 14 chlorophylls (green) (8 chlorophyll A and 6 chlorophyll B), 2 lutein (black), 1 violaxanthin (violet), and 1 neoxanthin (orange). The magnesium ions of the chlorophylls are represented as violet spheres. (**B**) RMSD of the ring portion of the fourteen chlorophylls from CG molecular dynamics simulations, with (green square) and without (red square) the restraints on the chlorophylls. The standard deviation is also shown. RMSF visualized on the AA (**C**) and CG (**D**) protein backbone structures as color gradient. The comparison is also shown in Figure S9. The flexibility of loops and terminal regions (blue region) is consistent in the AA and CG simulations.

Light-harvesting complexes are cofactor-binding protein systems responsible for light absorption and transfer toward the reaction center, PSII [11]. Each LHCII monomer binds a total of 18 pigments: 6 B chlorophylls , 8 A chlorophylls, and 4 xanthophyll molecules (lutein 1 and 2, violaxanthin, and neoxanthin). These pigments are embedded within the protein matrix and are mainly coordinated by three transmembrane helices that form the common motif of the LHC structure [11,48] (See Figure 4A). Although the LHCII trimer is the most abundant form of this complex in the thylakoid, LHCII monomers can also exist under native conditions [49,50]. Here, we focus on the LHCII monomer to validate the stability of the protein and the flexibility of the cofactors using the newly parametrized CG topologies against the AA model in a protein environment embedded in a membrane. We used the LHCII monomer extracted from the LHCII-PSII super-complex structure (PDB = 5XNM) [14]. Despite existing AA MD results for the LHCII monomer [18], we conducted additional AA simulations using the GROMOS force field to ensure consistency between the AA and CG reference structures. The computational details of both AA and CG simulations are described in Section 3. Briefly, the LHCII structure, including all the bonded cofactors, was embedded in a POPC bilayer and solvated with water, with counterions added to neutralize the system. The equilibrated AA structure was then mapped into the CG representation. 

Figure 4A shows the structure of the LHCII protein and its cofactors. To maintain the secondary structure of the protein in the CG model, we added Go potentials [51,52] between the backbone beads, a method that has proven effective in simulations of LHCII with Martini 2 [27]. The Go potential still allows for the observation of conformational transitions in proteins, which are involved in many crucial processes, including non-photochemical quenching.

**Figure 5:**
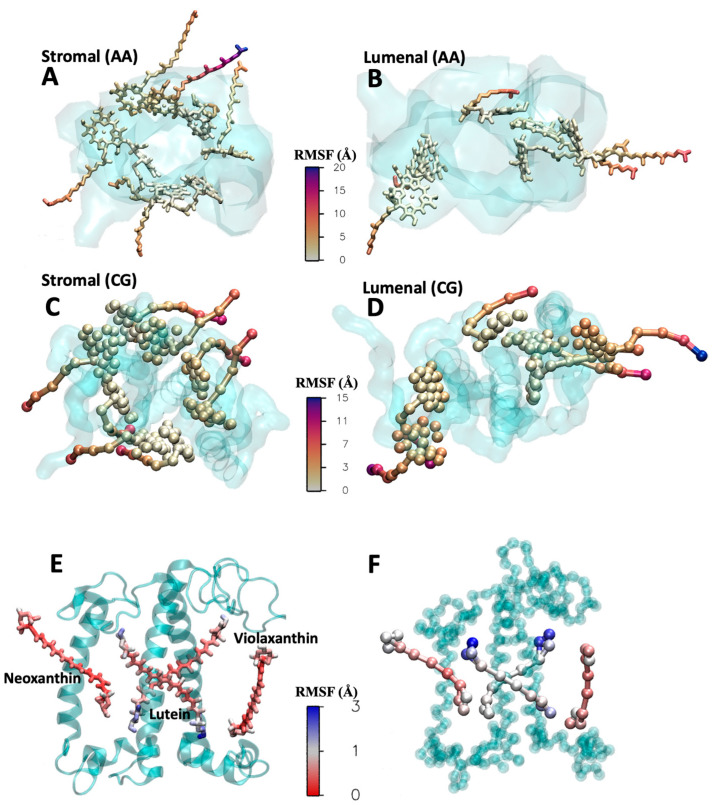
Comparison of cofactor dynamics in a protein environment. RMSF of the chlorophylls from the AA (**A**,**B**) and CG (**C**,**D**) simulations of the LHCII monomer. The protein is depicted as a cyan transparent surface. The chlorophylls are divided in two groups as stromal and luminal exposed. The chlorophylls are colored according to the RMSF, which is comparable in both the AA and CG. The aromatic core, buried into the protein, has very limited mobility, while the tails exposed to the membrane environment are much more flexible. RMSF of lutein, neoxanthin, and violaxanthin from the AA (**E**) and CG (**F**) simulations. The pigments are colored according to the RMSF. All of them are rather immobile (RMSF < 3 Å), with the heads showing higher mobility than the linker chain. The luteins from CG simulation are slightly more flexible than the AA counterparts.

A total of 3 microseconds of CG simulations was compared to 400 ns of AA simulations. In Figure 4C,D, we present the root mean square fluctuations (RMSF) of the LHCII monomer, visualized with a color gradient directly on the protein structures. The quantitative comparison is provided in Figure S9. The analysis reveals that in both AA and CG models, the three transmembrane helices exhibit the lowest RMSF, indicating their stability. However, the loops and termini situated at the membrane/water interface display higher flexibility in both the CG and atomistic simulations. The simulation is stable, with the cofactors remaining anchored to the protein without the use of restraints. In Figure 4B, we show the RMSD of the 14 chlorophylls, calculated using only the beads of the aromatic ring core. We compare the RMSD with (green squares) and without (red squares) positional restraints on the chlorophylls. In one simulation (green square), harmonic restraint bonds were added between the magnesium beads of the chlorophylls and the corresponding protein beads, with equilibrium values taken from atomistic simulations. The RMSD values are around 0.5–0.7 Å in both cases, with slightly more mobility observed in the chlorophylls without restraints. Nonetheless, the chlorophylls remain bonded to the side chain or the backbone of the coordinating amino acids. In Figure 5, we compare the RMSF of the chlorophylls in the CG and AA simulations, dividing the eight exposed to the stroma and the six exposed to the lumen for clarity. The aromatic core is very stable and consistent in the simulations at both resolutions. However, the tails show much more flexibility, especially for the peripheral chlorophylls tails exposed to the membrane environment. Additionally, the mobility of the tails is influenced by the structure of the monomeric protein we are considering. In the trimeric form, where each monomer is in contact with two homologous proteins, the mobility of the tails is significantly reduced because the surface exposed to the membrane is decreased [27]. In Figure 5E,F, the same type of plot, the RMSF, is shown for the xanthophylls violaxanthin, neoxanthin, and lutein in both the CG and AA simulations. No restraints were applied to the xanthophylls positions. In general, the heads are more flexible compared to the linker chains at both resolutions. The flexibility of lutein is slightly higher in the CG simulation compared to the atomistic one, although the difference is in the order of 1 Å. Meanwhile, the RMSF of neoxanthin and violaxanthin shows comparable mobility in both the CG and AA simulations.

### 2.4. Cofactors Embedded in the LHCII-PSII Super-Complex

**Figure 6:**
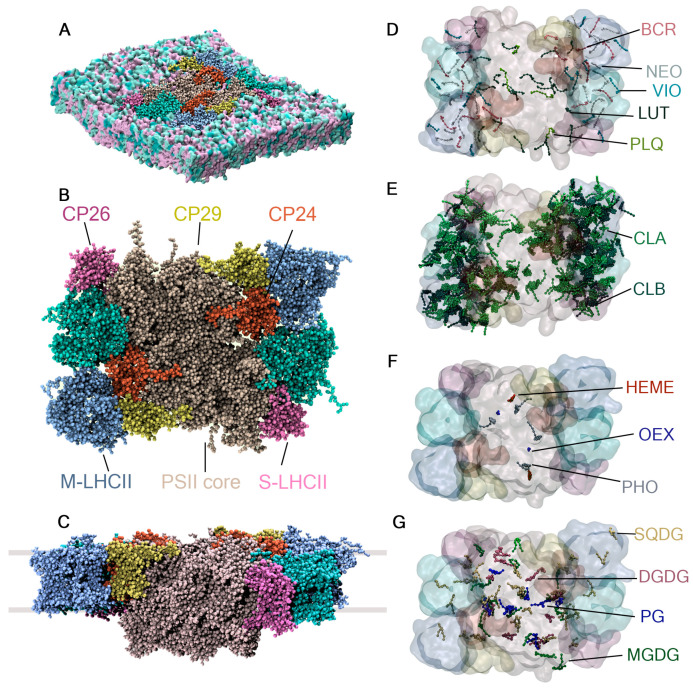
System setup for the PSII-LHCII super-complex. (**A**) PSII-LHCII super-complex model consisting of 56 protein chains and 514 co-crystallized cofactors embedded in the thylakoid membrane of plants. (**B**) Top view and (**C**) lateral view, with grey lines indicating the boundaries of the thylakoid membrane. The cofactors in the model are categorized into (**D**) carotenoids and plastoquinone, (**E**) chlorophylls, (**F**) low-abundance cofactors, and (**G**) thylakoid lipids.

To assess the behavior of the carotenoids, plastoquinone, and chlorophylls in a complex environment, a CG model of the C2S2M2-type PSII-LHCII super-complex from Pisum sativum (PDB ID: 5XNL) [14] embedded in the native thylakoid membrane of plants was built, as shown in Figure 6. The C2S2M2 super-complex contains two core subunits (C2), two strongly bound LHCII trimers (S2), and two moderately bound LHCII trimers (M2), as well as peripherally bound complexes CP24, CP26, and CP29. Previous simulations at the microsecond scale had studied the LHCII complex [27], the PSII dimer with Martini 2 [26], and recently, the C2S2 super-complex at the all-atom level with an amber force field [53]. A CG model of the C2S2M2 super-complex will enable the study of this system over longer timescales and the interaction of photosynthetic subunits that have not been co-crystallized with the super-complex, such as the photosynthetic subunit S (PsbS) [54].

The PSII-LHCII super-complex model consists of 56 protein chains, 24 of which have unique protein sequences. It also contains 514 co-crystallized cofactors of 15 different types: 216 A chlorophylls, 98 B chlorophylls, 32 luteins, 16 violaxanthins, 16 neoxanthins, 24 beta carotenes, 4 plastoquinones, 2 hemes, 2 oxygen-evolving complexes (OEX)—also known as CaMn4O5 clusters—and 2 A pheophytins (PHO). The model includes 49 co-crystallized lipids found in the thylakoid membrane of plants: 36 PG (16:0/16:0), 8 SQDG (16:0/16:0), 1 MGDG (18:0/18:0), and 10 DGDG (18:0/18:0). The details of the system’s construction, simulation settings, and treatment of the interactions between protein chains and cofactors are described in Section 3.

In the super-complex model, the co-crystallized thylakoid lipids, plastoquinone, and carotenoids—lutein, violaxanthin, beta-carotene, and neoxanthin—are free to diffuse in or out of the super-complex. In contrast, the OEX complex, heme, and pheophytin A are bound to the protein with elastic bonds. The chlorophylls are stabilized within the super-complex through magnesium–amino acid interactions and interactions between chlorophylls, also described by elastic bonds.

**Figure 7:**
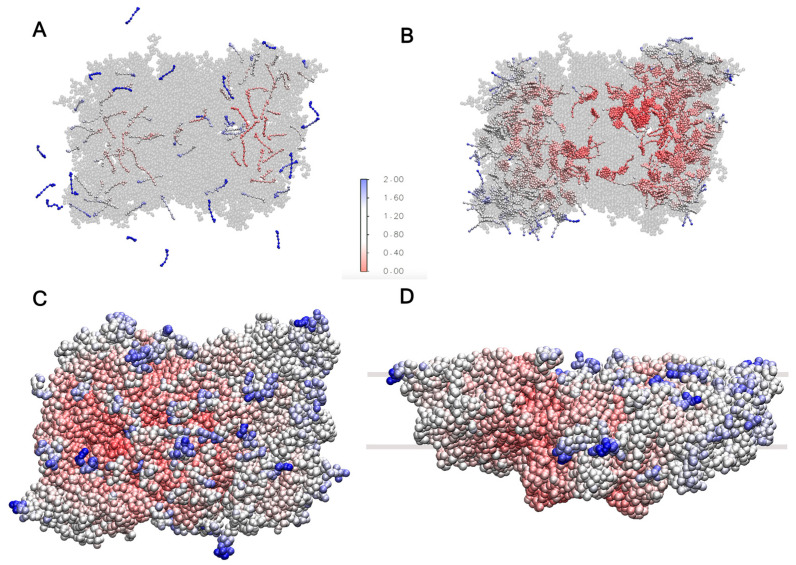
RMSF for (**A**) carotenoids and plastoquinone, where some of them diffuse outside the super-complex. (**B**) RMSF of chlorophylls A and B, showing high flexibility in the CP29 subunit and LHCII monomers. (**C**,**D**) RMSF of protein chains, indicating higher mobility in solvent-exposed regions and areas near the thylakoid membrane. In all panels, blue colors indicate high mobility and red colors low mobility.

On the (sub)microsecond scale, the freely moving cofactors should diffuse in and out of the super-complex [24,25]. To assess this behavior, the system was simulated for 1100 ns as described in Section 3. The RMSF over the last 1000 ns for the carotenoids and plastoquinone is shown in Figure 7A. The RMSF of the mobile cofactors is found to be highest in the outer regions of the LHCII trimers. By the end of the analyzed trajectory, nine of these cofactors had exited the super-complex. Additionally, the figure shows that the cofactors outside the protein environment can adopt parallel or inclined orientations with respect to the membrane plane due to their rotation within the thylakoid membrane, a phenomenon also observed in the POPC membrane model (Figure 3).

Chlorophylls A and B represent almost half of the co-crystallized cofactors. To assess their dynamics, the RMSF was calculated and is shown in Figure 7B. The chlorophylls located inside the CP29 subunit and the LHCII monomers, which are not directly bound to the core complex and are located in the outer regions of the super-complex, exhibit the highest flexibility due to their proximity to the membrane environment. This trend is also observed for the protein chains, as shown in Figure 7C,D, demonstrating that the solvent-exposed regions and areas in closer contact with the thylakoid membrane have higher mobility. The RMSF trends of the protein chains agree with the reported trends of the PSII dimer in Martini 2 [26] and the C2S2 super-complex simulated with atomistic detail [53].

An interesting case among the mobile cofactors is plastoquinone, which participates in the electron transport chain and can diffuse within both the PSII complex [25] and the cytochrome b6f complex [55]. As shown in previous simulations with the Martini 2 model, inside the PSII complex, plastoquinone enters through one of three channels, temporarily resides in the exchange cavity, is reduced to plastoquinol, and then exits the complex to continue the electron transport process in photosynthesis [25]. Capturing this phenomenon is relevant to evaluating the diffusion of cofactors within protein pores. In the current, still preliminary simulation, the timescale is too short to obtain meaningful statistics on plastoquinone/ol exchange, but plastoquinone was observed to diffuse around inside the exchange cavity during the 1100 ns simulation. 

## 3. Methods

### 3.1. Parametrization 

The development of coarse-grained (CG) models for the cofactors begins by deriving a set of preliminary bonded interactions from the distributions obtained in the mapped atomistic (AA) simulations. As previously mentioned, we used reference AA model trajectories based on the GROMOS force field produced by our group [31]. These preliminary models were then simulated at the CG level in dodecane, and their bond, angle, and dihedral profiles were compared to the mapped AA counterparts. This process was repeated until a satisfactory match was achieved. The computational parameters used for the CG simulations are consistent across all cofactors. The molecule was placed in a 5 × 5 × 5 nm^3^ box, which was then solvated with Martini dodecane. After minimization and equilibration steps, the system was simulated for 500 ns. The temperature and pressure were maintained at 298 K and 1.0 bar using a velocity rescale thermostat (time constant 1.0 ps) [56] and a Parrinello–Rahman barostat (time constant 12 ps) [57]. Electrostatic interactions were handled with a plain cut-off approach (cut-off at 1.1 nm). A time step of 20 fs was used for beta-carotene, violaxanthin, neoxanthin, and lutein, while chlorophyll A, chlorophyll B, and heme were stable with a time step of 10 fs. All simulations were conducted using GROMACS 2021.5 software [58]. Nonbonded interactions, determined by the selection of bead types, were derived from previously established Martini 3 models [28,32]. The accuracy of these parameters was assessed by estimating the water–octanol partition coefficient (log *P*) for each compound and comparing these values to experimental log *P* values. The solvent-accessible surface area (SASA) was used to estimate whether the chosen mapping of the cofactor molecule produced a reasonable shape and size at the coarse-grained level. This analysis was performed using the built-in *gmx sasa* tool in GROMACS 2021.5 software.

### 3.2. Free Energy and Partition Coefficient Calculations

The estimation of the water–octanol partitioning coefficient (log *P*_W/OCT_) was performed using the thermodynamic integration (TI) approach as implemented in the free energy code of GROMACS. Each molecule was placed in a cubic box containing water and hydrated octanol, with the water percentage at 10%. The interactions between the solute molecule and the solvent were switched off in 21 or 41 steps (“windows”), following a coupling parameter λ. The simulation was run for 8 ns at each λ value. The ΔG values were evaluated using Bennett’s acceptance ratio [59] (*gmx bar* tool). Soft-core potentials were applied with sc-α = 0.5 and sc-p = 1. The temperature was set to 300 K. The free energy of transferring a molecule from water (W) to octanol (Oct) was calculated as follows:

ΔG_W/Oct_ = ΔG_W/vacuum_ − ΔG_Oct/vacuum_ (1)

where Δ*G*_*W*/*vacuum*_ and Δ*G*_*Oct*/*vacuum*_ are the free energies obtained from the TI for both solvents. From this free energy, the partition coefficient (log *P*_W/Oct_) can be calculated:

(2) $$log\;P=\frac{-\Delta G\;}{ln\left(10\right)\;R\;T\;}$$

where *ln*(10) is the natural logarithm of 10, *R* is the gas constant, and *T* is the simulation temperature.

### 3.3. Cofactors in Membrane

Four molecules of carotenoids and xanthophylls (beta-carotene, violaxanthin, neoxanthin, lutein, and plastoquinone) were placed in a 128-lipid DPPC bilayer and solvated with Martini water molecules. The systems were built using the INSANE script [60]. Following minimization and a 1 ns equilibration, each system was simulated for 8 microseconds with a 20 fs time step. The temperature was maintained at 324 K (10 degrees above the phase transition temperature of DPPC) using a velocity rescale thermostat with a 1.0 ps time constant [56]. A semi-isotropic Parrinello–Rahman barostat was used to maintain a target pressure of 1 bar, with a time constant of 12 ps [57]. Electrostatic interactions were treated using the reaction field approach with a 1.1 nm cut-off and a shifted van der Waals potential, also with a 1.1 nm cut-off, using the Verlet cut-off scheme [61]. Electron densities were calculated along the Z-axis of the box, summing together the electrons of the atoms mapped to one CG bead and using the GROMACS analysis tool *g_density*. 

### 3.4. LHCII 

*All-atom MD simulation.* The structure of the LHCII, extracted from the PSII-LHCII super-complex structure (PDB ID: 5xnm) [14], was used for the initial starting configuration. The protein with all the embedded cofactors (8 chlorophyll A, 6 chlorophyll B, 2 lutein, violaxanthin, lutein, and DPPG) was embedded in POPC lipid bilayer and solvated with ~3660 water molecules with a 150 mM NaCl concentration. The gromos53a6 force field was used, because it is compatible with the parameters of the cofactors that we used as reference for the CG parametrization [31,33]. After the minimization, the system was equilibrated in two steps:

(1) NVT equilibration. Here, no pressure coupling was applied. Long-range interactions were evaluated using particle mesh Ewald summation with a 12 Å cut-off in real space [62]. Lennard–Jones (LJ) interactions were truncated at 12 Å with an atom-based force switching function, which starts to be effective at 10 Å. The integration time step was set at 2 fs for 200 ps. Bonds involving hydrogen atoms were constrained using the LINCS algorithm [63]. A velocity rescale thermostat [56] was used to maintain the temperature at 300 K. Position restraints were applied on the protein.
(2) NPT equilibration. Here, A semi-isotropic Parrinello–Rahman [57] barostat with a reference pressure of 1 bar and isothermal compressibility of 4.5 × 10^−5^ bar^−1^ was used to maintain the pressure of the system, while a Nosé–Hoover chain thermostat was used to maintain the temperature at 300 K [64]. The simulation was run for 1 ns.

Finally, the production run was performed for 400 ns with these last computational parameters but removing the restraints on the protein structure. 

*Martini 3 simulation.* An equilibrated structure of the protein with the embedded cofactors from the previous simulation was extracted and mapped into the CG representation. The cofactor-binding protein was then embedded into a POPC bilayer and solvated with Martini water using the INSANE script [60]. The Go potential was applied to ensure the stability of the protein’s secondary structure [51]. The system was simulated for 3 microseconds with a time step of 10 fs using the following computational parameters: electrostatic interactions were treated using the reaction field approach with a 1.1 nm cut-off [61], a velocity rescale thermostat [56] with a 1.0 ps time constant and a target temperature of 300 K, and a Parrinello–Rahman barostat to maintain a target pressure of 1 bar with a time constant of 12 ps [57]. We conducted simulations both with and without restraining the bonds between the 14 chlorophylls and the coordinating protein beads. Specifically, for each chlorophyll, a harmonic restraint can be applied between the magnesium bead and the coordinating protein bead. The parameters for the restraints are as follows: the equilibrium distance (extracted from atomistic dynamics) and the force constant (1000 kJ mol^−1^ nm^−2^). Both types of simulations proved to be stable, and the chlorophylls did not detach from the protein.

### 3.5. PSII-LHCII

*Protein model preparation.* The crystal structure of the C2S2M2-type PSII-LHCII super-complex from Pisum sativum, PDB ID 5XNL [14], formed the basis of our model. PDB 5XNL was chosen instead of 5XNM because it contains one extra protein chain and the overall resolution is higher. Any missing residues were constructed with pymol [65] and optimized with Modeller [66]. Most of the unique protein chains have less than 24 missing residues, mainly at the flexible terminals. The chain X has 44 unmodelled residues in the n terminal and 3 in the c terminal and homology modeling is not possible; instead, the Alpha fold 2 structure UniprotID Q8VYY1 was used [67,68]. The reconstructed chains were fitted to the crystal structure using USalign. To convert the chains to CG resolution, Martinize2 [69] was used with the ElNeDyn approach to stabilize the secondary structure with standard values (cut-off = 0.9 nml force constant Fc = 500 kJ mol^−1^ nm^−2^). The cofactors were mapped to the coarse-grained resolution using pycgtool [70].

*Inclusion of cofactors.* Due to their low abundance in the PSII-LHCII super-complex, the OEX complexes and pheophytin were modeled qualitatively, using the previous Martini 2 simulations of the photosystem II as a guideline [26]. The CaMn4O5 clusters were modeled by 4 beads separated by 6 bonds with a force constant of 10,000 and charge of 2+ and an equilibrium distance given by the mapped crystal structure. Both pheophytin A and chlorophyll A share the same basic chlorin ring structure, but pheophytin a lacks the central magnesium ion present in chlorophyll A, having two hydrogen atoms in its place. Thus, for pheophytin A, we used the same parameters as for chlorophyll A, removing the magnesium bead and setting the charge to zero; the constraints were changed to bonds with force constants of 50,000 kJ mol^−1^ nm^−2^ to improve the stability of the central ring. The Fe(II) ions and bicarbonate ions were not included in this version of the model. 

The chlorophyll A, chlorophyll B, heme, and pheophytin a were bound to the protein chains with elastic bonds with force constants of 300 kJ mol^−1^ nm^−2^, (1000 kJ mol^−1^ nm^−2^ in case of pheophytin a). The residues to attach the cofactors were chosen based on the information available in the PDB file of the crystal structure [14], which was visualized in the PDBSum server [71]. For some cofactors that had missing non-hydrogen atoms, the models available in the PDB database were fitted to the resolved structure, selecting the atoms that had the best fitting and minimizing the RMS value using MDAnalysis.

*Embedding in thylakoid membrane.* Our model of the thylakoid membrane of plants has the composition described by Sakurai et al. [72] and was previously characterized using the Martini 2 model by van Eerden et al. [23]. The PG, MGDG, DGDG, and SQDG thylakoid lipids are part of the alpha version of the new Martini 3 lipid parameter set and will be discussed in a separate publication. The CG chains and cofactors, together with the thylakoid lipids, were inserted in a simulation box of size 35 × 35 × 16.7 nm using INSANE [60]. The system contained 176,123 beads, including 897 thylakoid lipids per leaflet, 99,687 water beads, 976 neutralizing sodium ions, and a salt concentration of 0.15 M NaCl. 

*Simulation settings*. The Gromacs 2021.3 package was used to perform the simulations [58]. The initial structure was minimized using the steepest descent algorithm for a maximum number of 50,000 steps. The system was simulated using the reaction field approach to describe long-range electrostatics with cut-off values of 1.1 nm for short-range Coulomb and van der Waals interactions [61]. To control temperature and pressure, the v-rescale thermostat with a coupling parameter of 1.0 ps [56] and the Berendsen semi-isotropic barostat [73] at 1 bar with a τ_p_ of 4 ps and compressibility of 3.0 × 10^−4^ bar^−1^ were used.

Before production, the system was gradually equilibrated in an NVT ensemble, applying position restraints on the protein backbone beads, and gradually increasing the temperature from 200 K to 300 K during 7 steps at reduced time steps of 0.001–0003 ps for a total of 35 ns. Subsequently, the position restraints were removed, and the system was simulated at 300 K with a timestep of 0.003 ps for 3 ns and then with a timestep of 0.01 ps for 100 ns in the NPT ensemble. The production run consisted of 1100 ns with a timestep of 0.01 ps under the NPT ensemble at 298.15 K. The c-rescale barostat was used to control the pressure and the rest of the settings were the same as the equilibration, taking into consideration the recommended neighboring list setting, updating every 20 steps [74]. For the RMSF analysis, the gmx rms tool was used, disregarding the first 100 ns. The super-complex was made whole over the periodic box using the mdvwhole tool [75].

## 4. Conclusions

In this work, we focused on the Martini 3 parametrization of the cofactors associated with light harvesting and PSII core complexes, i.e., beta-carotene, violaxanthin, neoxanthin, lutein, plastoquinone, chlorophylls A and B, and heme. The development and validation of these CG models has proven successful in accurately capturing the structural and dynamic properties of these molecules in different environments. The consistent fragmentation of xanthophylls and carotenoids, including beta-carotene, violaxanthin, neoxanthin, lutein, and plastoquinone, reflected their molecular structures well. The CG distributions closely matched those from atomistic simulations, despite minor volumetric underestimations typical of the Martini model. For chlorophylls and heme groups, characterized by complex aromatic ring systems and charged centers, the CG approach maintained molecular symmetry and charge distribution effectively. CG simulations within a DPPC lipid bilayer and the LHCII monomer demonstrated the CG models’ capability to replicate the dynamic behavior and organization of cofactors in membrane and protein environments. Consistent root mean square fluctuations between AA and CG models confirmed the stability and reliability of the CG models. The ability of CG models to capture hydrophobicity trends and spatial organization of cofactors in lipid membranes, along with their stability within protein environments, underscores their applicability in studying complex biological systems. 

In addition, we used these cofactor parameters to construct a CG model of the entire LHCII-PSII super-complex at the Martini 3 level, within the native thylakoid membrane. We considered DPPC or POPC membrane models, but the complex nature of glycolipids in the thylakoid membrane impacts the stability between the various protein components of the super-complex [24,26,27]. Moreover, the presence of multiple assembled protein units also affects the mobility of the cofactors [27]. These analyses will be the subject of future studies. Creating such a large and complex system would open the door to studying the functional mechanisms, stability, and photoprotection of photosynthesis, including non-photochemical quenching (NPQ) [13,76,77]. The hypothesis is that there is an aggregation between various monomeric LHCII subunits, which can be accessible to simulate with the CG model of the super-complex [12,76,78]. 

## Data Availability

Data are contained within the article and Supplementary Materials. The structures and parameters of the cofactors will be also uploaded and available in the Martini Database [79] (https://mad.ibcp.fr, accessed on 15 July 2024).

## Supplementary Materials

The following supporting information can be downloaded at: https://www.mdpi.com/article/10.3390/ijms25147947/s1.
