# Supplementary material for: Martini 3 Coarse-Grained Model for the Cofactors Involved in Photosynthesis
Source: Int J Mol Sci. 2024 Jul 20;25(14):7947. doi: 10.3390/ijms25147947 (PMC11277265; doi:10.3390/ijms25147947)
Supplement: Supplementary file 1 [file ijms-25-07947-s001.zip › ijms-3092061-supplementary.pdf]

Supporting Information

Martini 3 Coarse-Grained Model for the Cofactors Involved in Photosynthesis

Maria Gabriella Chiariello, Rubi Zarmiento-Garcia, Siewert-Jan Marrink *

Groningen Biomolecular Sciences and Biotechnology Institute, University of Groningen, Nijenborgh 7, 9747 AG Groningen, The Netherlands.

* Corresponding author

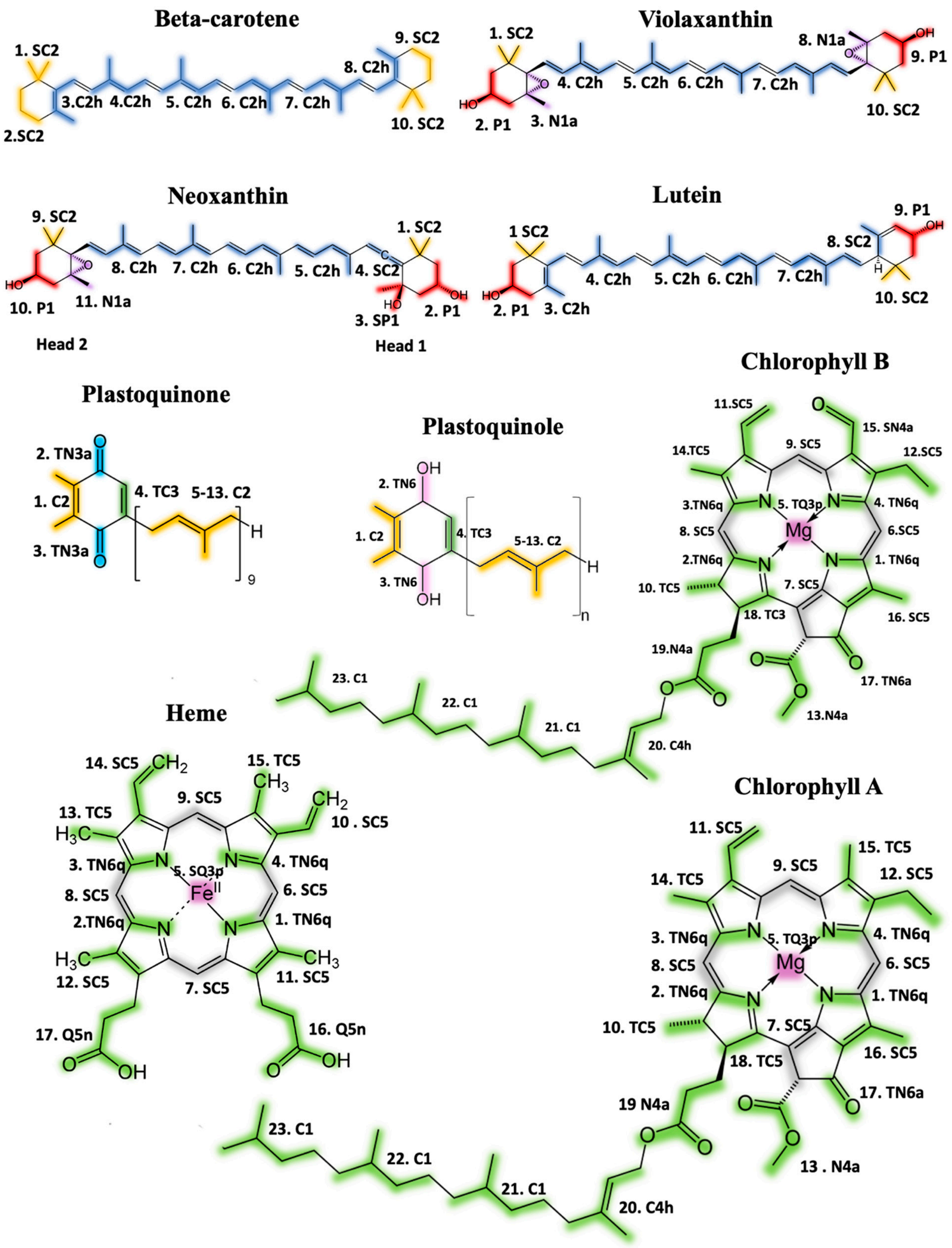

Figure S1. Molecular structures and CG mapping scheme of the cofactors parametrized in this work: Beta-carotene, Violaxanthin, Neoxanthin, Lutein**,** Plastoquinone, Plaqtoquinol, Chlorophyll A and B and Heme group. The atoms of the atomistic structure are mapped into CG beads.

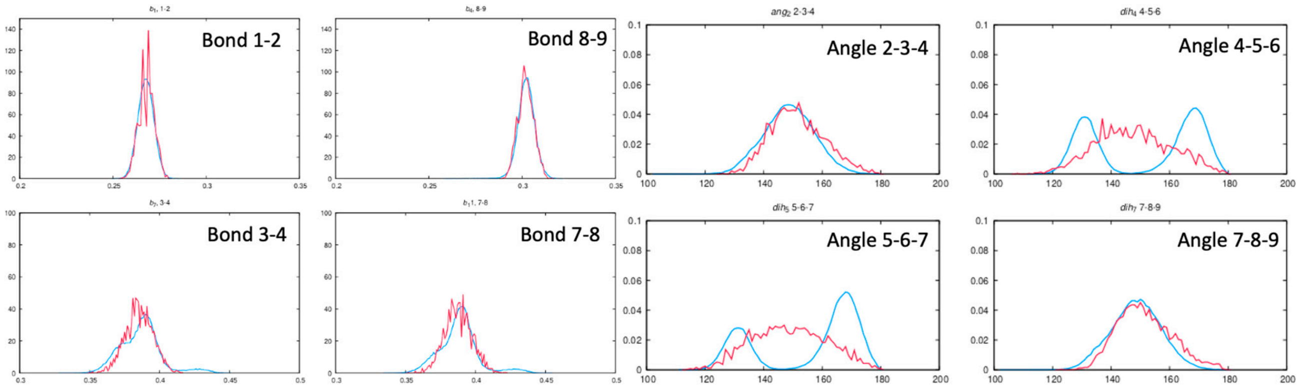

Figure S2. Comparison between the AA (blue) and CG (red) distributions of selected bonds and angles parameters of **beta-carotene**. The bead numbers are labelled in Figure S1.

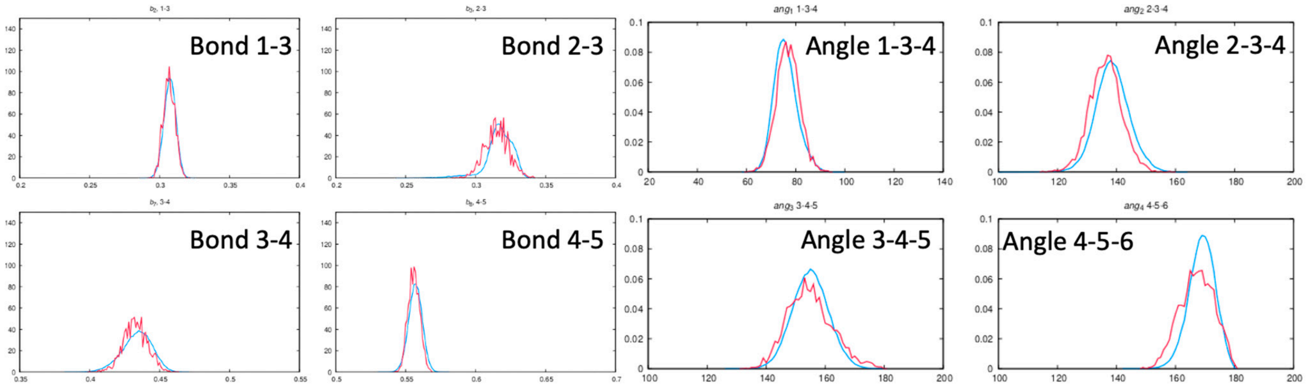

Figure S3. Comparison between the AA (blue) and CG (red) distributions of selected bonds and angles parameters of **violaxanthin.** The bead numbers are labelled in Figure S1.

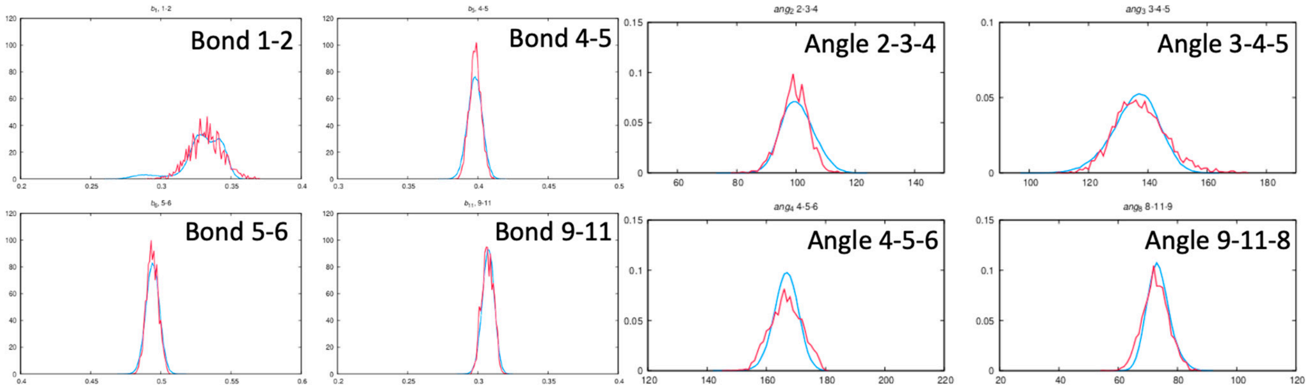

Figure S4. Comparison between the AA (blue) and CG (red) distributions of selected bonds and angles parameters of **neoxanthin.** The bead numbers are labelled in Figure S1.

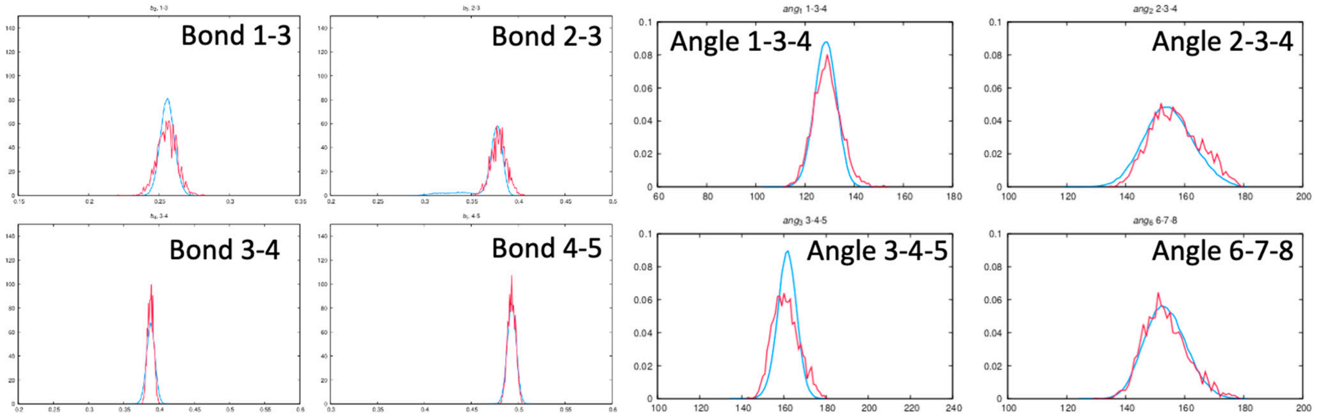

Figure S5. Comparison between the AA (blue) and CG (red) distributions of selected bonds and angles parameters of **lutein.** The bead numbers are labelled in Figure S1.

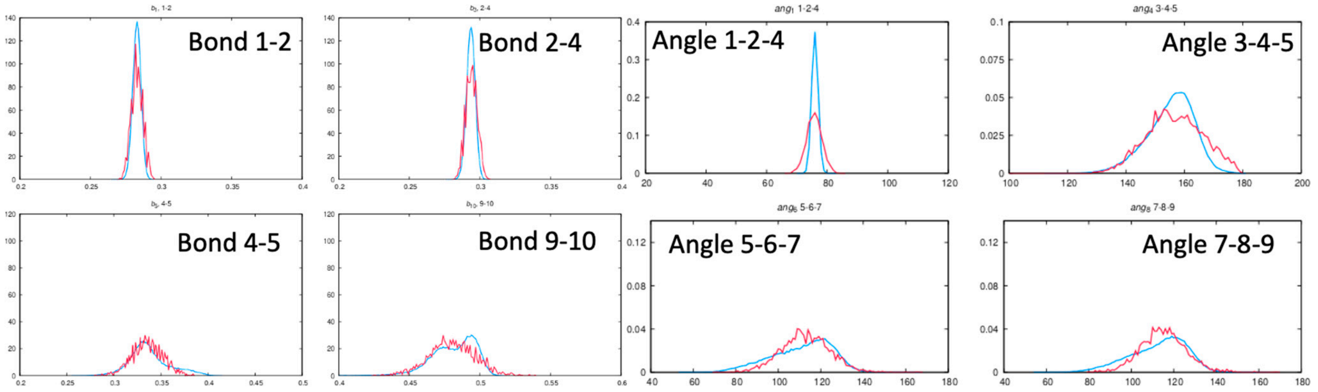

Figure S6. Comparison between the AA (blue) and CG (red) distributions of selected bonds and angles parameters of **plastoquinone.** The bead numbers are labelled in Figure S1.

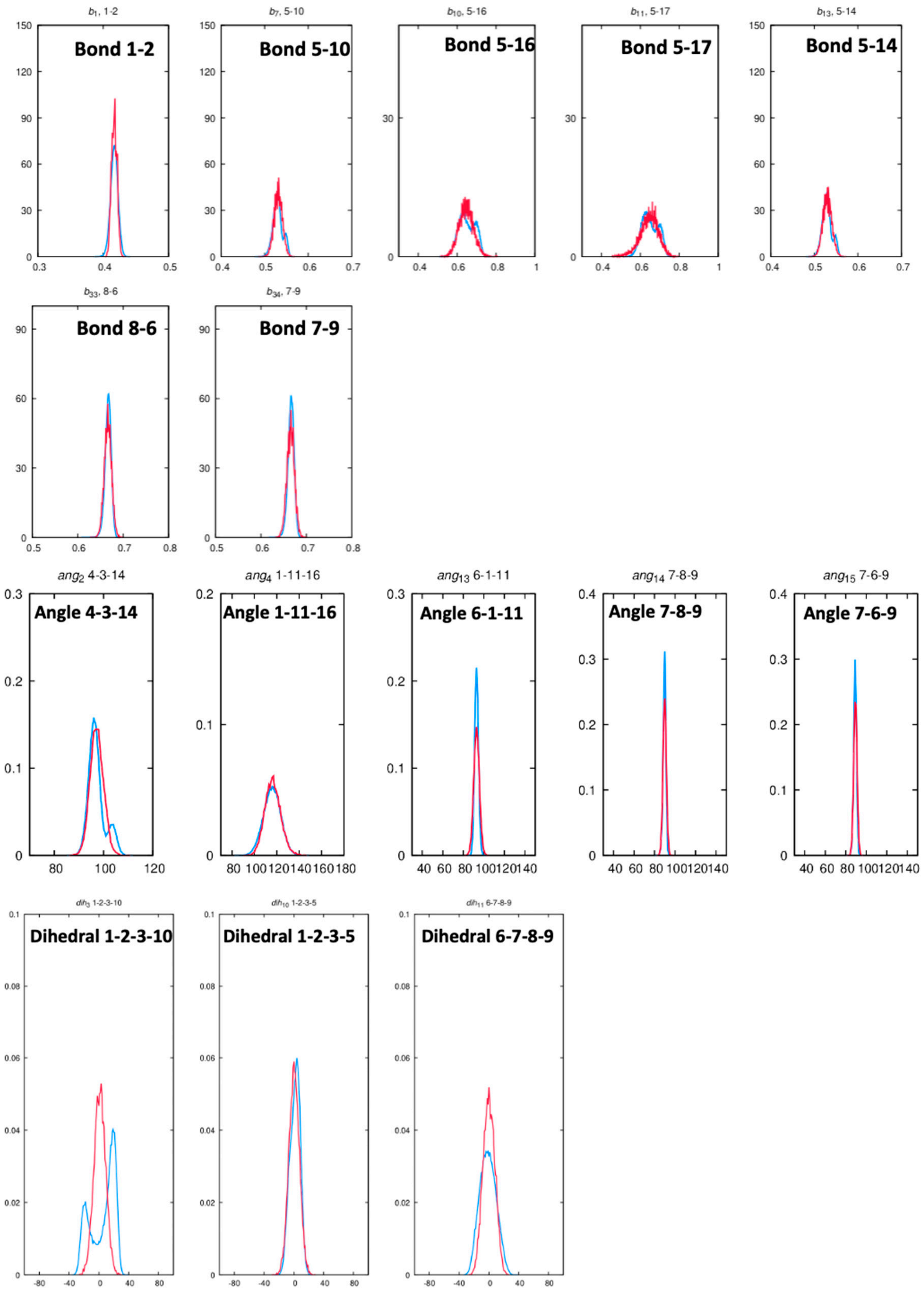

Figure S7. Comparison between the AA (blue) and CG (red) distributions of selected bonds and angles parameters of **heme.** The bead numbers are labelled in Figure S1.

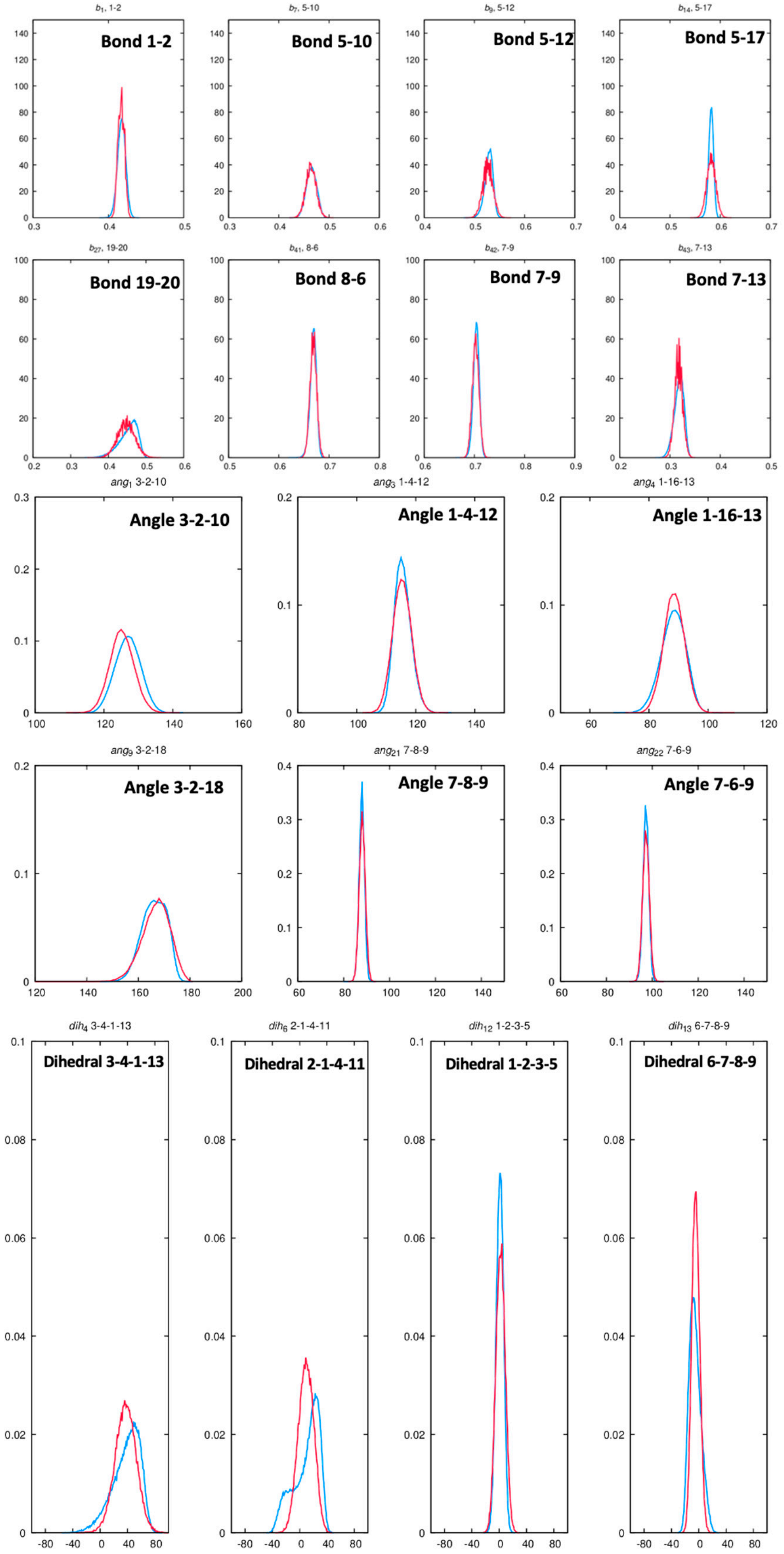

Figure S8. Comparison between the AA (blue) and CG (red) distributions of selected bonds and angles parameters of **chlorophyll A.** The bead numbers are labelled in Figure S1.

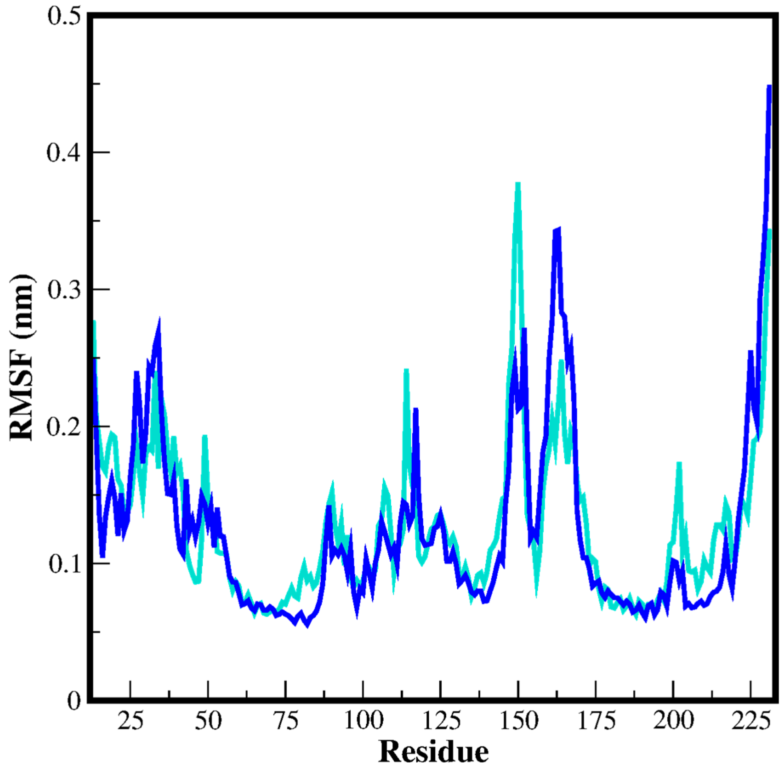

Figure S9. Comparison of the root mean square fluctuations (RMSF) computed for the all-atom simulation (cyan) and Martini 3 model for the LHCII backbone.
